# Palliative chemotherapy or best supportive care? A prospective study explaining patients' treatment preference and choice

**DOI:** 10.1038/sj.bjc.6601445

**Published:** 2003-12-09

**Authors:** C G Koedoot, R J de Haan, A M Stiggelbout, P F M Stalmeier, A de Graeff, P J M Bakker, J C J M de Haes

**Affiliations:** 1 Department of Medical Psychology, Academic Medical Center, PO Box 22700, 1100 DE, Amsterdam, The Netherlands; 2 Department of Clinical Epidemiology and Biostatistics, Academic Medical Center, Amsterdam, The Netherlands; 3 Department of Clinical Decision Making, Leiden University Medical Center, Leiden; 4 Department of Internal Medicine, University Medical Center, Utrecht; 5 Department of Internal Medicine, Academic Medical Center, Amsterdam, The Netherlands

**Keywords:** palliative chemotherapy, best supportive care, decision-making, treatment preference, treatment choice

## Abstract

In palliative cancer treatment, the choice between palliative chemotherapy and best supportive care may be difficult. In the decision-making process, giving information as well as patients' values and preferences become important issues. Patients, however, may have a treatment preference before they even meet their medical oncologist. An insight into the patient's decision-making process can support clinicians having to inform their patients. Patients (*n*=207) with metastatic cancer, aged 18 years or older, able to speak Dutch, for whom palliative chemotherapy was a treatment option, were eligible for the study. We assessed the following before they consulted their medical oncologist: (1) socio-demographic characteristics, (2) disease-related variables, (3) quality-of-life indices, (4) attitudes and (5) preferences for treatment, information and participation in decision-making. The actual treatment decision, assessed after it had been made, was the main study outcome. Of 207 eligible patients, 140 patients (68%) participated in the study. At baseline, 68% preferred to undergo chemotherapy rather than wait watchfully. Eventually, 78% chose chemotherapy. Treatment preference (odds ratio (OR)=10.3, confidence interval (CI) 2.8–38.0) and a deferring style of decision-making (OR=4.9, CI 1.4–17.2) best predicted the actual treatment choice. Treatment preference (total explained variance=38.2%) was predicted, in turn, by patients' striving for length of life (29.5%), less striving for quality of life (6.1%) and experienced control over the cause of disease (2.6%). Patients' actual treatment choice was most strongly predicted by their preconsultation treatment preference. Since treatment preference is positively explained by striving for length of life, and negatively by striving for quality of life, it is questionable whether the purpose of palliative treatment is made clear. This, paradoxically, emphasises the need for further attention to the process of information giving and shared decision-making.

Eventually, almost 50% of cancer patients will have metastatic disease (Dutch Cancer Foundation, 1999). Still, making treatment decisions in palliative cancer treatment is a complex task for patients as well as physicians. This is particularly true because there is no evident ‘best’ option. This paper reports a prospective study on patients having to make a choice between palliative chemotherapy and best supportive care. Palliative chemotherapy aims at the alleviation of symptoms or postponing future symptoms of the disease and thus maintaining or enhancing quality of life. Survival gains, if present, are modest. For an individual patient, it is uncertain whether symptom alleviation or survival gain can be achieved while side effects are likely to occur. Best supportive care is the alternative option and aims at enhancing quality of life by the relief of symptoms once they occur. At the same time, limited survival gain, even a few extra months, may be important from the patient's perspective.

Whether a patient with metastatic cancer should be treated with palliative chemotherapy or choose best supportive care is therefore not always evident. Clinically seen, individual patients may benefit from palliative chemotherapy. However, contradicting evidence can be found in the literature on the effects of chemotherapy on the quality of life. There are studies in which chemotherapy appears to enhance patients' quality of life (Thatcher *et al*, 1997; Stockler *et al*, 1998; Tannock, 1998; Geels *et al*, 2000; Doyle *et al*, 2001). Ramirez *et al* (1998) found that only 25% of the patients benefited from chemotherapy in her study, and there are several studies in which no improvement or even deterioration of quality of life was reported (Quantin *et al*, 2000; van Andel *et al*, 2000; Barras *et al*, 2001; Schiller *et al*, 2001). Values of patients thus become more important. Knowledge of the factors upon which patients' preferences or the patients' actual choice are based is necessary in guiding the patient through the decision-making process.

Four factors influencing cancer patients' treatment choice have been distinguished by Richards *et al* (1995): patients' attitudes and beliefs, doctors' attitudes, the way information is presented and the nature of the risk and benefit involved in the different treatment options. Patients' attitudes and beliefs are thought to be based on previous experience, family, friends, media and other health-care professionals (Richards *et al*, 1995; Jansen *et al*, 2001). Doctors' attitudes are influenced by what they think is the aim of the therapy; relief of symptoms, extending life and giving hope (Maher *et al*, 1992). Several studies have looked at the influence of the way information is presented to patients (McQuellon *et al*, 1995; Yellen and Cella, 1995; Flood *et al*, 1996; Mazur and Hickam, 1997; O'Connor, 1998). It was found that the framing of information affects patients' choices (Siminoff and Fetting, 1991; McQuellon *et al*, 1995; Flood *et al*, 1996; Mazur and Hickam, 1997; O'Connor, 1998). The nature of the risk and benefit of the different treatment options can indeed influence the patient's treatment choice, as shown in several studies concerning early disease (Richards *et al*, 1995). However, the relative value attached to treatment options in palliative care, such as chemotherapy and supportive care, is less well documented (Richards *et al*, 1995).

In contrast to the lack of studies on treatment choice, several studies have been performed on the nature and background of treatment preference (Cassileth *et al*, 1980; McNeil *et al*, 1982; Llewellyn-Thomas *et al*, 1995; Flood *et al*, 1996; Brundage *et al*, 1997; Mazur and Hickam, 1997; Lindley *et al*, 1998; Silvestri *et al*, 1998; Stalmeier and Bezembinder, 1999; Stiggelbout and de Haes, 2001). In the literature, the patient's preference for treatment is seen and used as an indication for their actual treatment choice. However, patients' actual treatment choice and their intended choice or treatment preference are not necessarily the same, since treatment preference is usually assessed in patients or subjects who are not actually facing the choice investigated.

Several factors are found to affect treatment preference. Firstly, framing influences treatment preferences, that is, whether outcomes are presented in terms of survival or in terms of death, and whether or not medical uncertainties are explicitly mentioned (McQuellon *et al*, 1995; Flood *et al*, 1996; Mazur and Hickam, 1997; O'Connor, 1998). Secondly, disease experience seems to be of influence: patients are more willing than the general public or medical staff to accept toxic treatment (Slevin *et al*, 1990; Degner and Sloan, 1992; Brundage *et al*, 1997). Thirdly, various demographic variables such as age, living with others and having children may also have an impact on treatment preferences of patients (Kiebert *et al*, 1994; Unic *et al*, 1998).

It is generally assumed that patients form preferences and make decisions after they have been informed by their physician. Preferences for treatment are thus usually assessed after patients have received information concerning their treatment options (McNeil *et al*, 1982; Flood *et al*, 1996; O'Connor, 1998; Lindley *et al*, 1998; Silvestri *et al*, 1998). However, the patient, being a modern consumer of health care, is not a tabula rasa. The notion that medical professionals are the first to discuss certain topics and that patients depend fully on their information and advice, is no longer necessarily true. Patients have information from family, friends, media or the Internet. Based on these sources of information, most patients may already have an idea about chemotherapy and whether they want it for themselves or not. To what extent these preferences translate into a treatment decision is unknown.

To understand the decision-making process in palliative chemotherapy, we investigated the actual treatment choice of patients with metastatic cancer. Owing to its indicative value, we prospectively studied their strength of preference for palliative chemotherapy or best supportive care in cancer patients before they consulted their medical oncologist. Finally, we investigated whether this treatment preference, patient or disease characteristics, quality of life, attitudes or preference for information and participation in decision-making were predictive of the actual treatment choice.

Our conceptual model is presented in Figure 1

**Figure 1:**
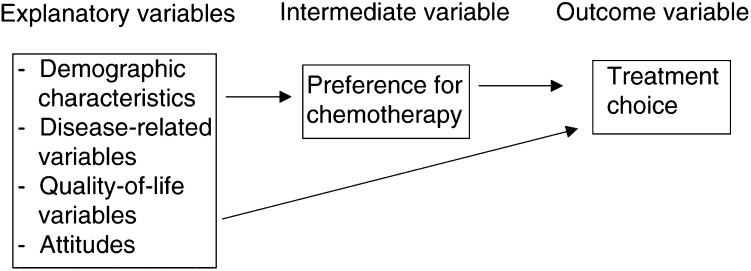
Conceptual model (explanatory variables, intermediate variable and outcome variable).

.

## PATIENTS AND METHODS

This prospective study took place between June 1998 and April 2000. Patients with various types of metastatic cancer, aged above 18 years, able to understand and speak Dutch, referred because palliative chemotherapy had become a treatment option were eligible for the study. The patients were approached as soon as an appointment with their medical oncologist was scheduled. After informed consent was given, an appointment was made for an interview prior to the first consultation with their medical oncologist. After the decision concerning palliative chemotherapy or best supportive care had been made, the treatment choice was checked. To facilitate participation, the interview was held at the patient's home.

Medical oncologists (*n*=37) from three academic and seven nonacademic hospitals participated in the study. They completed a disease-related checklist for each patient after they had seen the patient during consultation. The oncologist informed the researchers about the treatment decision as soon as it was made. This was checked with the patient within 3 weeks. Ethical approval was obtained from the ethics committee of each participating hospital.

### Measures

#### Dependent variables

For ethical reasons, we did not want to interfere with the expectations or knowledge of patients before the consultation took place. Therefore, we first asked them in an open-ended question as to what treatment they expected their physician would propose in the upcoming consultation. Next, the strength of preference for chemotherapy was assessed in those patients who expected their medical oncologist to propose chemotherapy as one of the options with a single question on a seven-point Likert scale (‘strong aversion to chemotherapy’ to ‘strong preference for chemotherapy’). If a patient did not mention chemotherapy, the part of the questionnaire concerning chemotherapy was skipped.

Patients' choices were assessed, as described, after the actual decision was made.

#### Predictor variables

The independent variables, that is (1) socio-demographic patient characteristics, (2) disease-related variables, (3) quality-of-life indices and (4) attitudes were assessed at baseline (1, 3, 4) or obtained from the oncologist (2).

Demographic variables included gender, age, marital status, having children and educational level. Disease-related variables comprised type of cancer and performance status, the Karnofsky index (Karnofsky and Burchenal, 1949)

Quality of life was measured with the Rotterdam Symptom Checklist (RSCL) (de Haes *et al*, 1990). The RSCL is a validated, cancer-specific questionnaire and covers physical distress (22 items, Cronbach's *α*=0.80 in the present study), psychological distress (eight items, *α*=0.89) and the daily activity level (eight items, ADL; *α*=0.86). The item scores range from 1 to 4. Sum scores were transformed to the same scale. Low scores on the physical and psychological distress scale reflect a better quality of life, whereas low scores on the activity level scale indicate a worse quality of life.

Several attitudes were assessed. The Cancer Locus of Control Scale (CLOC) covers the attitude of patients towards the origin and the course of their illness (Pruyn *et al*, 1988; Watson *et al*, 1990). It consists of three subscales: internal control over the disease process (*α*=0.74), internal control over the cause of the disease (*α*=0.64) and religious control (*α*=0.88). Item scores range from 1 to 4, a higher score indicating a higher level of control.

The patient's decision style was assessed with The Michigan Assessment of Decision Style (MADS) (Pierce, 1993, 1995). The MADS is developed for decision-making in early-stage breast cancer, and validated. It was translated into Dutch for the current study by two bilingual individuals, using a forward–backward translation procedure. It covers: (1) avoidance (four items, e.g. ‘I prefer not knowing the possibility that unexpected things could happen to me’, *α*=0.55); (2) deferring responsibility (three items, e.g. ‘I would follow the recommendations of my physician’, *α*=0.66); (3) information seeking (four items, e.g. ‘I would spend as much time as I could gathering information’, *α*=0.83); and (4) deliberation (five items, e.g. ‘I would carefully consider the risks of each option as I was making a choice’, *α*=0.73). The scale scores were linearly converted to a 0–100 scale, with higher scores indicating a higher level of avoidance, deferring of responsibility, information seeking and deliberation.

Patients' attitudes towards striving for length (quantity) (four items, *α*=0.75) or quality of life (four items, *α*=0.69) were assessed by the ‘QQ-Questionnaire’ (QQQ) (Stiggelbout *et al*, 1996). High scores on the quantity or quality scale indicate the importance of length and quality of life, respectively.

Preference for information was measured using a 10-point rating scale, ranging from score 1 (wanting as little information as possible) to score 10 (wanting all the information there is) (Blanchard *et al*, 1988). Patients' preference for participation in decision-making was assessed on a five-point rating scale ranging from giving the physician full responsibility for decision-making (score 1), to the patient wanting to take this role (score 5) (Sutherland *et al*, 1989; Degner and Sloan, 1992).

### Statistical analyses

Univariate associations between baseline preference for treatment and demographic characteristics, disease-related factors, quality of life and attitudes were established with Pearson's product moment correlations and point biserial correlation coefficients.

Significant predictors of treatment preference (with *P* set at <0.25) identified from the univariate analyses were then entered into a multiple linear regression analysis, with a forward selection strategy, using the F-statistic with *P*=0.05 as the criterion level for selection in the multivariate analyses.

Possible predictors of treatment choice were analysed with the *χ*^2^ statistic and expressed in crude relative risk (RR) estimates with their 95% confidence intervals (CI). Continuous variables were dichotomised by using the median split method. For baseline treatment preference, however, a content-related split was made (either having or not having a preference for chemotherapy). Due to skewness, the preference for information scale scores was recoded as ‘do not prefer as many details as possible’ (1–9), and ‘prefer as many details as possible’ (10). Additionally, all variables univariately associated with treatment choice (*P*-value set at <0.25) were entered in a logistic regression model to assess their independent prognostic value for treatment choice. Effect sizes were expressed in odds ratios (ORs) (with their 95% CI). Calibration of the regression model was assessed with the Hosmer–Lemeshow goodness-of-fit test. In this test, a high *P*-value indicates that the model is acceptable. All analyses were performed in SPSS (version 10.0.7).

## RESULTS

### Patients

Of 242 patients, recruited over a 2-year period, 35 patients were not eligible because they were treated with curative intent or were not offered the choice of palliative chemotherapy. Of the remaining 207 patients, 140 patients were interviewed (68% response). Reasons for not willing to participate at baseline were as follows: poor physical condition (*n*=39), reported psychological distress (*n*=2), time constraints (*n*=10) or unspecified (*n*=16). The actual treatment decision could be confirmed by 131 patients. Nonresponse was due to being too ill (*n*=6) or death (*n*=3).

Patient characteristics are presented in Table 1

**Table 1:** Patient characteristics (*n*=140)

	**N**	**%**
**Socio-demographic**		
*Gender*		
Male	85	61
Female	55	39
*Age (years)*		
26–50	27	19
51–60	40	29
61–70	45	32
71–82	28	20
*Children*		
Yes	119	85
No	21	15
*Education*		
Primary school	30	22
High school	81	58
College or higher	28	20

**Disease-related**		
*Type of cancer*		
Breast cancer	14	10
Head and neck cancer	22	16
Gastric-intestinal cancer (sum)	68	49
Oesophagus	9	6
Stomach	7	5
Colon	28	20
Pancreatic	9	6
Rectum	14	10
Non-small-cell lung cancer	9	6
Other	27	19
*Performance status*		
100	53	39
90	48	35
80	23	17
70	9	7
60	3	2

**Quality of life**		
Physical distress	M=1.50; s.d.=0.34	
Psychological distress	M=1.74; s.d.=0.64	
ADL activity level	M=3.70; s.d.=0.52	

**Attitudes**		
*Locus of control*		
Disease process	M=3.18; s.d.=0.60	
Cause of the disease	M=1.80; s.d.=0.54	
Religious	M=2.13; s.d.=1.16	
*Decision-making style*		
Information seeking	M=2.66; s.d.=0.93	
Deliberation	M=3.98; s.d.=0.56	
Avoidance	M=2.43; s.d.=0.65	
Deferring	M=3.98; s.d.=0.58	
Striving for length of life	M=3.35; s.d.=1.13	
Striving for quality of life	M=3.74; s.d.=0.99	
Preference for information	M=8.91; s.d.=1.99	
Preference for participation	M=3.07; s.d.=0.79	

a Due to missing values, the numbers do not always add to 140. s.d.=standard deviation.

. More than half of the sample was male (61%); the mean age was 60 years (s.d. 11.6).

### Treatment preference and actual choice

Most patients (*n*=114; 81%) expected that their medical oncologist would propose chemotherapy. Subsequently their preference for chemotherapy could be assessed. Patients who did not answer that they expected the physician to propose chemotherapy (*N*=26) were older (*P*<0.01).

**Figure 2:**
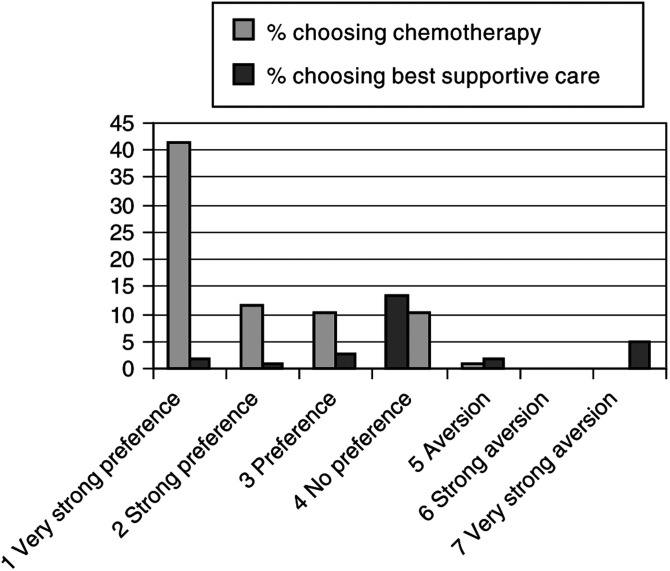
Preferences for either palliative chemotherapy or best supportive care and the Patients' actual treatment choice (*n*=1).

The distribution of the baseline treatment preference is presented in Figure 2

. Most patients (68%) favoured chemotherapy at baseline. The majority of these had a very strong preference for chemotherapy. Eventually 78% chose to undergo chemotherapy. In Figure 2 the actual choice is shown, per treatment preference category. The original treatment preference and the eventual treatment choice turned out to be related. Almost all patients who preferred chemotherapy before they visited their medical oncologist chose chemotherapy after they had discussed their treatment. Approximately half of the patients (56%) who had no clear treatment preference before they met with their oncologist chose chemotherapy. Of those who had an aversion towards chemotherapy (7%), almost all chose best supportive care, eventually.

### Explaining treatment preference at baseline

In Table 2

**Table 2:** Relation (univariate) between patient characteristics at baseline and their strength of preference for palliative chemotherapy (*n*=114)

	**Preference for chemotherapy^a^**	
	***r*^b^**	***P***
**Socio-demographic**		
Gender	−0.09	0.32
Age (older)	−0.20	0.04
Children^c^	0.06	0.52
Education	−0.05	0.63

**Disease-related**		
*Type of cancer*^c^		
Breast	0.10	0.28
Head/neck	0.15	0.12
Gastric-intestinal cancer (sum)	−0.10	0.28
Lung	0.04	0.67
Performance status	0.02	0.81

**Quality of life**		
Physical distress	0.05	0.58
Activity level (ADL)^d^	−0.11	0.23
Psychological distress^e^	0.11	0.24

**Attitudes**		
*Locus of control*		
Disease process^f^	0.25	<0.01
Cause of the disease^f^	0.16	0.10
*Decision style*		
Information seeking^g^	−0.01	0.95
Deliberation^g^	−0.05	0.63
Avoidance^g^	−0.03	0.76
Deferring^g^	0.30	<0.001
Striving for length of life	0.55	<0.001
Striving for quality of life	−0.51	<0.001
Preference for information	0.15	0.10
Preference for participation	−0.18	0.06

a Positive signs indicate a stronger preference for chemotherapy.

b PMCCs.

c Point biserial correlation.

d Higher score less limited.

e Higher score more distress.

f High scores indicate high control.

g High scores indicate a more active style.

, the relation between preference for chemotherapy and the explanatory variables is presented.

Younger patients had a stronger preference for chemotherapy. Neither other demographic variables nor disease-related or quality-of-life-related variables were significantly related to the Patients' preference for palliative chemotherapy, although some attitudes were. High levels of internal control concerning the disease process, having a stronger deferring decision style, striving for length of life and having a low preference for participating in the decision-making were associated with a stronger preference for chemotherapy. Striving for quality of life was negatively related to the strength of preference for chemotherapy.

Multivariate analyses indicated that Patients' preferences for chemotherapy were best explained by striving for length of life (*β*=0.38, partial *R*^2^=29.5%), whereas less striving for quality of life added 6.1% (*β*=−0.29) to the explained variance. Feeling internal control concerning the cause of the disease added an additional percentage of 2.6% (*β*=0.16).

### Explaining treatment choice

In Table 3

**Table 3:** Relation between patient characteristics and preference for chemotherapy at baseline, and the Patients' actual treatment choice (*n*=131)

	**Treatment choice (N)**				
**Background factors**	**Chemotherapy**	**Best supportive care**	**Crude RRb**	**95% CI**	***P***
**Socio-demographic**					
*Sex*					
Female	42	9			
Male	60	20	1.10	0.92–1.31	0.32
*Age (years)*					
>61	46	15			
⩽61	56	14	0.94	0.78–1.13	0.53
*Children*					
Yes	87	25			
No	15	4	0.98	0.76–1.27	0.90
*Education*					
High	26	8			
Low	75	21	0.98	0.79–1.21	0.84

**Disease-related**					
*Type of cancer*					
Breast cancer					
Yes	12	1			
No	90	28	1.21	1.00–1.46	0.19
Head/neck cancer					
Yes	15	5			
No	87	24	0.96	0.73–1.26	0.74
Gastric-intestinal cancer (sum)					
Yes	47	16			
No	55	13	0.92	0.77–1.11	0.39
Lung cancer					
Yes	8	1			
No	94	28	1.15	0.90–1.48	0.41
Performance status	53	21			
⩽90	48	8	0.84	0.70–1.00	0.06
>90					

**Quality of life**					
*Physical distress*					
High (>1.47)	49	12			
Low (⩽1.46)	53	17	1.06	0.88–1.27	0.53
*Activity level (ADL)*					
High (>3.76)	62	15			
Low (⩽3.75)	40	14	1.09	0.90–1.32	0.38
*Psychological distress*					
High (>1.51)	49	15			
Low (⩾1.50)	53	14	0.97	0.81–1.16	0.73

**Attitudes**					
*Locus of control*					
Disease process					
High control (>1.5)	54	13			
Low control (⩽1.5)	48	15	1.06	0.88–1.27	0.54
Cause of the disease					
High control (>3.37)	44	14			
Low control (⩽3.37)	58	15	0.95	0.79–1.15	0.62
*Decision-making style*					
Information seeking					
High (⩾2.75)	50	17			
Low (<2.75)	52	12	0.92	0.77–1.10	0.36
Deliberation					
High (⩾4.0)	55	19			
Low (<4.0)	47	10	0.90	0.75–1.08	0.27
Avoidance					
High (⩾2.5)	55	18			
Low (<2.5)	47	11	0.93	0.78–1.11	0.44
Deferring					
High (⩾4.0)	41	3			
Low (<4.0)	61	26	1.33	1.13–1.56	<0.01
Striving for length of life					
Length more important (>3.4)	5				
Length less important (⩽3.4)	43	24	1.44	1.18–1.74	<0.001
Striving for quality of life					
Quality more important (>3.4)	45	22			
Quality less important (⩽3.4)	57	7	0.75	0.62–0.91	<0.01
Preference for information					
Strong (10)	69	16			
Weak (0–9)	33	13	1.13	0.92–1.39	0.21
Preference for participation					
Strong (3–5)	89	27			
Weak (1, 2)	13	2	0.89	0.71–1.11	0.38
Preference for chemotherapy					
Preference (1–3)	67	6			
No preference (4–7)	15	18	2.02	1.38–2.95	<0.001

a Due to missing values, the numbers do not always add to 131.

b RR>1 indicates a stronger likelihood to choose chemotherapy. RR=relative risk; CI=confidence interval.

, the univariate relations between explanatory factors and treatment choice are shown. None of the socio-demographic variables was significantly related to the treatment chosen, nor were disease-related variables and quality of life; however, attitudes were. Having a deferring decision style, striving for more length of life and less for quality, as well as having a strong preference for palliative chemotherapy at baseline were all significantly predictive of an eventual choice for palliative chemotherapy.

From Table 4

**Table 4:** Factors explaining treatment choice (*n*=131)

	**ORb**	**95% CI**	***P***
Performance status	2.5	0.64–7.55	0.21
Deferring decision style	4.9	1.40–17.18	0.01
Striving for length of life	1.7	0.43–6.96	0.44
Striving for quality of life	1.1	0.29–4.16	0.89
Preference for information	2.5	0.74–8.20	0.14
Preference for chemotherapy	10.3	2.80–37.96	<0.001

a Due to cells with zero respondents, having breast cancer was left out of the Logistic regression analysis.

^b^Multivariate logistic regression analysis. Hosmer and Lemeshow test: *P*=0.73. OR=odds ratio; CI=confidence interval.

, presenting the multivariate analysis explaining Patients' actual treatment choices, it appears that only ‘baseline preference for chemotherapy’ and ‘a deferring decision style’ explain the eventual treatment choice. Patients with a strong baseline preference for chemotherapy were substantially more likely to choose chemotherapy (OR=10.3). Patients who had a deferring decision style were also more likely to start chemotherapy (OR=4.9). The Hosmer–Lemeshow goodness-of-fit statistic (*P*=0.73) indicated that the multiple regression model was well calibrated.

## DISCUSSION

The most remarkable finding in our study is, in our view, that the Patients' preference for chemotherapy as assessed before they met with their medical oncologist, most strongly predicted their eventual treatment choice. Patients seem to make up their minds about starting or forgoing palliative chemotherapy before they are informed by their medical oncologist and, thus, outside the consultation room. A conclusion therefore must be that what was said during the consultation did not change much of the patient preference and eventual choice.

Another striking finding is that the initial treatment preference was strongly explained by striving for length of life. Oncological consensus is that the expected outcomes of palliative chemotherapy and best supportive care differ little in survival in most tumour types (Glimelius *et al*, 2001; Ragnhammer *et al*, 2001). Still, patients chose to be treated with chemotherapy, seemingly clinging to the hope that this chemotherapy would extend their life duration. They may thus not have received some basic information or, alternatively, not have heard it. Alternatively, patients did not want to hear this information or did not believe it and chose palliative chemotherapy, being fully aware of its limited possibilities regarding lengthening of life. Additionally, both a preference for chemotherapy and choosing palliative chemotherapy were negatively associated with striving for quality of life. Bearing in mind the purpose of palliative chemotherapy, the enhancement or maintenance of quality of life (Porzsolt, 1993; Porzsolt and Tannock, 1993), one would expect striving for quality of life to be positively associated with preference and choice for palliative chemotherapy. Therefore, one can question whether the purpose of palliative chemotherapy has been clearly explained to and is understood by all patients.

Of the patients in our study, 68% had a preference for chemotherapy, before they had received information from their medical oncologist about treatment options; 78% of the patients decided to undergo palliative chemotherapy eventually. In other words, in absolute sense the impact of the consultation on the treatment chosen is limited. The relationship between preference for treatment and the actual treatment choice has not been investigated thoroughly (Yellen and Cella, 1995; Jansen *et al*, 2001). In retrospect, it is less surprising that the treatment preference at baseline is so closely related to the actual treatment choice. For patients and physicians, choosing an active treatment option, that is, palliative chemotherapy, seems obvious. Best supportive care is often perceived as ‘doing nothing’ (Charles *et al*, 1998). For medical oncologists, the patient's wish is an important determinant of their own preference for treatment (Charles *et al*, 1998; Koedoot *et al*, 2002). Moreover, they also prefer to ‘do something’, that is, offering chemotherapy, rather than offering best supportive care (de Haes and Koedoot, 2003). Thus, Patients' and oncologists' treatment preferences seem to coincide, making a choice for chemotherapy more likely.

Treatment choice was also predicted by having a deferring style of decision-making. Patients having such decision-making style were more likely to undergo chemotherapy than others. Since medical oncologists, being experts in systemic treatment, want to offer a treatment, and spend more time explaining chemotherapy than explaining best supportive care, they may convey the suggestion that they prefer palliative chemotherapy (Koedoot *et al*, 1996). It is then likely that a deferrer, who would like to lean on the oncologist's advice, is inclined to choose chemotherapy. The absence of an association between a deferring decision-making style and baseline preference for chemotherapy is not surprising, since at baseline deferrers are not yet aware of the oncologist's preference. They have not had an opportunity to defer the decision to their oncologist yet.

Some limitations of our study have to be mentioned. Firstly, there is most likely a referral bias. Patients, who visit a medical oncologist, often have a positive attitude towards treatment already. Indeed, two-thirds of our patients preferred to be treated with palliative chemotherapy before the consultation. Secondly, for ethical reasons, only patients who mentioned that they expect their physician to offer them palliative chemotherapy were included in the analyses. In doing so, we could have created a selection bias. Patients who did not expect their oncologist to offer them chemotherapy were older than those who expected their oncologist to offer chemotherapy (*t*=−2.51, df=138, *P*=0.01). Still, age appeared to have no influence on the actual treatment choice. Therefore we conclude that no important selection bias is at stake. Thirdly, the content of the information given by the referring specialists is unknown. Therefore, it would be of interest to look at Patients' preferences even before they are referred to a medical oncologist.

In conclusion, our results suggest that physicians could take these preconsultation ideas and preferences into account when providing information to patients. The information-giving process should then address the limited survival benefit of palliative chemotherapy and the possibility of best supportive care. Explicit attention may also have to be given to the ‘natural’ inclination of both physician and patient to ‘do something’, as it may be questioned whether the modest survival gain, in palliative chemotherapy, is to be considered worthwhile. Moreover, treating with palliative chemotherapy may be less than good quality-of-life care (Singer *et al*, 1999). Thus, the need for open physician–patient communication and shared decision-making with regard to treatment in this phase of the disease is stressed again. Finally, it might be necessary to discuss explicitly the attitudes of patients towards chemotherapy during the consultation in order to trace possible misconceptions. Our findings, thus, point to the need for a model of shared decision-making, in which different treatment options are explained, Patients' attitudes and beliefs are investigated and the actual treatment choice is the outcome of a joint decision-making process (Charles *et al*, 1999). Especially because the available treatment options are equivalent in palliative treatment, this concept deserves extensive attention when proposing palliative chemotherapy or best supportive care.
